# Comments on Ekino *et al.* Cloning and Characterization of a Unique Cytotoxic Protein Parasporin-5 Produced by *Bacillus thuringiensis* A1100 Strain. *Toxins* 2014, *6*, 1882–1895

**DOI:** 10.3390/toxins7124865

**Published:** 2015-11-27

**Authors:** Leopoldo Palma

**Affiliations:** 1 Facultad de Ciencias Agrarias, Universidad Nacional del Litoral, Esperanza 3080, Santa Fe, Argentina; lpalma@fca.unl.edu.ar; Tel.: +54-9-3496-418223; 2 Consejo Nacional de Investigaciones Científicas y Técnicas, Ciudad Autónoma de Buenos Aires C1083ACA, Argentina

Ekino *et al.* [1] reported the cloning and characterization of a novel cytotoxic protein (Parasporin-5) produced by *Bacillus thuringiensis* strain A1100. The 33.8-kDa inactive precursor protein exhibited strong cytocidal activity upon proteinase K activation against several mammalian (cancer) cell lines, and showed slight homology with Cry and aerolysin-type β-pore-forming toxins. Most research concerning parasporins has mainly been performed in order to demonstrate their use as potential therapeutic agents against cancer, but they are lacking additional research supporting the absence of activity against invertebrates; especially, taking into account that these toxins are not expected to evolve to kill cancer cells. Therefore, it is reasonable to think that they should have another (unknown) target in nature. Despite the fact that in this work, the authors demonstrated the toxic activity of this protein against several types of cancer cells, further complementary studies against a minimum number of insects would be of great interest in order to determine the potential insecticidal activity of this protein and understand its natural role. For example, Palma and collaborators [2] reported the molecular and insecticidal characterization of a novel Cry-related protein closely related to parasporins 2 and 4, (Cry41Aa1 and Cry41Ab1). This protein did not show any toxic activity against five species of Lepidoptera but, after more extensive testing, this protein was found to demonstrate a specific toxic activity against the green-peach aphid *Myzus persicae*. Nowadays, parasporin proteins are commonly known in the literature to be produced by “non-insecticidal” *B. thuringiensis* strains and because they exhibit significant and preferential cytocidal activity against cancer cells of various origins [3]. However, the absence of insecticidal activity deserves to be more deeply investigated since a single *B. thuringiensis* toxin has shown to have a narrow host range while, in general, they are active against a wide range of invertebrates [4,5]. Therefore, the determination of the activity against a minimum number of insects (preferably from different taxonomic orders) is highly desirable and might change the non-insecticidal concept we currently have about parasporins proteins produced by non-insecticidal *B. thuringiensis* strains.
